# A novel diagnostic tool reveals mitochondrial pathology in human diseases and aging

**DOI:** 10.18632/aging.100546

**Published:** 2013-03-24

**Authors:** Morten Scheibye-Knudsen, Karsten Scheibye-Alsing, Chandrika Canugovi, Deborah L. Croteau, Vilhelm A. Bohr

**Affiliations:** ^1^ Laboratory of Molecular Gerontology, National Institute on Aging, National Institutes of Health, Baltimore, MD 20892, USA; ^2^ Section of Genetics and Bioinformatics, IBHV, Faculty of Health and Medical sciences, University of Copenhagen, Denmark

**Keywords:** aging, mitochondria, progeria, bioenergetics, diagnostics, bioinformatics

## Abstract

The inherent complex and pleiotropic phenotype of mitochondrial diseases poses a significant diagnostic challenge for clinicians as well as an analytical barrier for scientists. To overcome these obstacles we compiled a novel database, www.mitodb.com, containing the clinical features of primary mitochondrial diseases. Based on this we developed a number of qualitative and quantitative measures, enabling us to determine whether a disorder can be characterized as mitochondrial. These included a clustering algorithm, a disease network, a mitochondrial barcode and two scoring algorithms. Using these tools we detected mitochondrial involvement in a number of diseases not previously recorded as mitochondrial. As a proof of principle Cockayne syndrome, ataxia with oculomotor apraxia 1 (AOA1), spinocerebellar ataxia with axonal neuropathy 1 (SCAN1) and ataxia-telangiectasia have recently been shown to have mitochondrial dysfunction and those diseases showed strong association with mitochondrial disorders. We next evaluated mitochondrial involvement in aging and detected two distinct categories of accelerated aging disorders, one of them being associated with mitochondrial dysfunction. Normal aging seemed to associate stronger with the mitochondrial diseases than the non-mitochondrial partially supporting a mitochondrial theory of aging.

## INTRODUCTION

Bona fide mitochondrial diseases represent a heterogeneous group of genetic syndromes with a combined incidence of around 1:5000 [1]. The clinical diversity represents a considerable diagnostic challenge for pediatricians often leading to a delay in diagnosis [1]. Because of the early onset and rapid progression of many of these diseases an early diagnosis will become increasingly important with the accelerating development of mitochondrial therapeutic strategies. Although the complete pathogenesis remains unknown, energy deficiency in affected tissues is believed to be the causative agent in most of these disorders [2].

Besides their cardinal role in ATP metabolism mitochondria are the main producers of endogenous oxidative radicals. These highly volatile species react with lipids, proteins and nucleic acids in their vicinity. The mitochondrial theory of aging states that an accumulation of damage to these macromolecules throughout the lifetime of an organism leads to cellular decay, loss of tissue homeostasis, and finally to death [3]. Multiple lines of evidence have corroborated this theory and suggested that mitochondrial maintenance may be important in promoting longevity and healthy aging [4-9]. Indeed, mitochondria have been implicated in most age related diseases such as neurodegeneration [10], cardiovascular disease [11] and diabetes [12]. If mitochondrial dysfunction is causative in aging, we would expect the accelerated aging disorders [13] to exhibit features of mitochondrial disease.

To investigate this, we compiled a database of the clinical parameters seen in mitochondrial diseases, www.mitodb.com. Based on this database we developed extensive bioinformatics tools to dissect whether a disease could be characterized as mitochondrial or not. These tools include three qualitative and two quantitative measures. Using these tools we identified a number of diseases as mitochondrially associated that had not previously been considered as mitochondrial. Recently a number of diseases, such as ataxia-telangiectasia, Cockayne syndrome, ataxia with oculomotor apraxia 1 (AOA1) and spinocerebellar ataxia with axonal neuropathy 1 (SCAN1) have been suggested to have mitochondrial dysfunction and these disorders were also identified by our tools [14-17]. With the validation of the tools we went on to investigate the mitochondrial involvement in a number of monogenic diseases. Interestingly, Parkinson's disease, Huntington's disease and amyotrophic lateral sclerosis all showed a substantial mitochondrial involvement. Further, when adding the accelerated aging disorders to the database two groups of progeria appeared; one group associated with chromosomal instability and another group clustered with mitochondrial diseases. Normal aging seemed to associate closer with the mitochondrial group in the clustering algorithm but showed mixed mitochondrial and non-mitochondrial values in the support vector machine and mitoscore. Taken together these findings indicate at least two separate causes of aging, one of them possibly being mitochondrial.

## RESULTS

### www.mitodb.com

Using various sources such as the United Mitochondrial Disease Foundation webpage (http://www.umdf.org), Pubmed (http://www.ncbi.nlm.nih.gov/pubmed) and the Online Mendelian Inheritance in Man database (http://www.omim.org) we identified 31 monogenic diseases that all have been characterized as mitochondrial describing a total of 1,265 patients (Supplementary table 1). To get as broad a spectrum of mitochondrial phenotypes as possible we added respiratory chain defects, fatty acid oxidation defects and other primary mitochondrial diseases. 25 non-mitochondrial monogenic diseases, used as controls, describing a total of 21,032 patients, were selected based on well established pathogenesis with no or very minimal involvement of mitochondria. A database, www.mitodb.com, was constructed by writing ~8,000 lines of code in the languages html and php and using the aforementioned data sources, referenced signs, symptoms, laboratory and paraclinical findings (collectively referred to as clinical parameters or parameters) were recorded for each disease by clinically experienced physicians. The prevalence of each parameter was listed based on what was reported in the most authoritative literature we could identify. The publication date was as current as possible (Supplementary figure 1). For any particular disease we used the largest cohorts we could identify. Case reports were excluded. If we used data from two or more studies the prevalence was the average of the combined number of patients. All the references including their abstracts, publication date, periodical and links to Pubmed can be found online at www.mitodb.com. Each disease was thus characterized by a number of clinical parameters with a prevalence from 0-100 percent. See supplementary figure 2 or www.mitodb.com for visual examples of how the diseases are represented in the database. We will present a number of figures that can be generated online at www.mitodb.com. We also recommend reading our tutorial on the following URL: www.mitodb.com/tutorial.html.

### Mitochondrial diseases form a distinct clinical group

**Figure 1:**
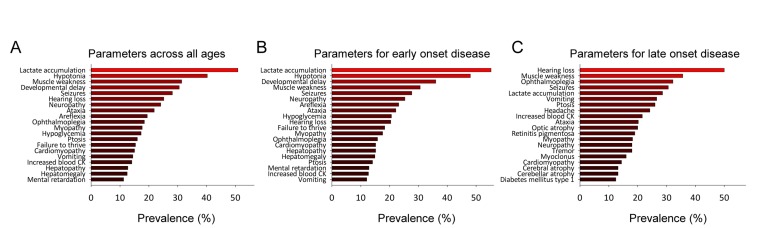
Mitochondrial diseases have a defined clinical spectrum (**A**) The top-20 clinical parameters seen in all mitochondrial diseases. (**B**) The top-20 clinical parameters seen in mitochondrial diseases with a mean age of onset before 20. (**C**) The top-20 clinical parameters seen in mitochondrial diseases with a mean age of onset after 20.

Based on the data we could now analyze the clinical parameters in the mitochondrial group by averaging the occurrence of a parameter across all the diseases in each group. This allowed us to investigate whether any particular parameter would be more prevalent among the mitochondrial diseases than among the non-mitochondrial diseases. Figure 1A shows the top 20 most prevalent parameters and supplementary figure 3 and 4 shows all the parameters in the mitochondrial group and the non-mitochondrial group, respectively. Our analysis shows that, out of 117 clinical parameters seen in mitochondrial diseases, lactate accumulation, hypotonia, muscle weakness, developmental delay and seizures are among the most common. Since it has been suggested that the clinical parameters of mitochondrial diseases depend upon the age of onset of the disease we investigated whether this was the case for our dataset. We identified the mean age of onset and standard deviation for each disease in the database (See supplementary table 1). Using this data we could then model the age of onset of each clinical parameter letting us identify the most common symptoms for diseases with onset before age 20 and most common symptoms for diseases with onset after age 20 (Figure 1B and C) and their evolution over time (Supplementary figure 5). Lactate accumulation, hypotonia, developmental delay, muscle weakness and seizures were the most prevalent parameters in the early onset diseases while hearing loss, muscle weakness, ophthalmoplegia, seizures and lactate accumulation were the most prevalent in the late onset diseases. Some differences in clinical parameters were thus found when comparing early onset to late onset disease. However, quantifying these differences as a Pearson coefficient resulted in a value of 0.5 indicating an overall similar phenotype in early and late onset diseases. In addition, there was no published data for the individual parameter onsets for every disease preventing us from using age of onset for further analysis. We therefore decided to continue our analysis without including age of onset.

By overlaying the prevalences of all the mitochondrial and non-mitochondrial parameters it became evident that some mitochondrial clinical parameters occurred at a high frequency indicating a high degree of similarity between the diseases in the mitochondrial group while there was much more heterogeneity amongst the clinical features in the non-mitochondrial disease group (Supplementary figure 6A). Based on this resemblance among the mitochondrial diseases, we investigated how these diseases would associate with each other when clustering them using the software cluster 3.0 [18]. Supplementary figure 6B shows an example of how some mitochondrial and some non-mitochondrial diseases cluster with each other based on clinical parameters. The clustering of the diseases is shown in the top, while the clinical parameters are shown on the right with the clustering of the parameters on the left. The tint of each square in the center of the cluster map reflects the prevalence of a parameter in the disease based on published literature. Interestingly, there seemed to be excellent separation between the mitochondrial and non-mitochondrial diseases. Supplementary figure 6C shows an overview of the cluster while a detailed look can be seen online at www.mitodb.com. In summary, we were able to use cluster analysis to compare the diseases in the database.

### The qualitative determination of a mitochondrial phenotype using mitodb.com

**Figure 2:**
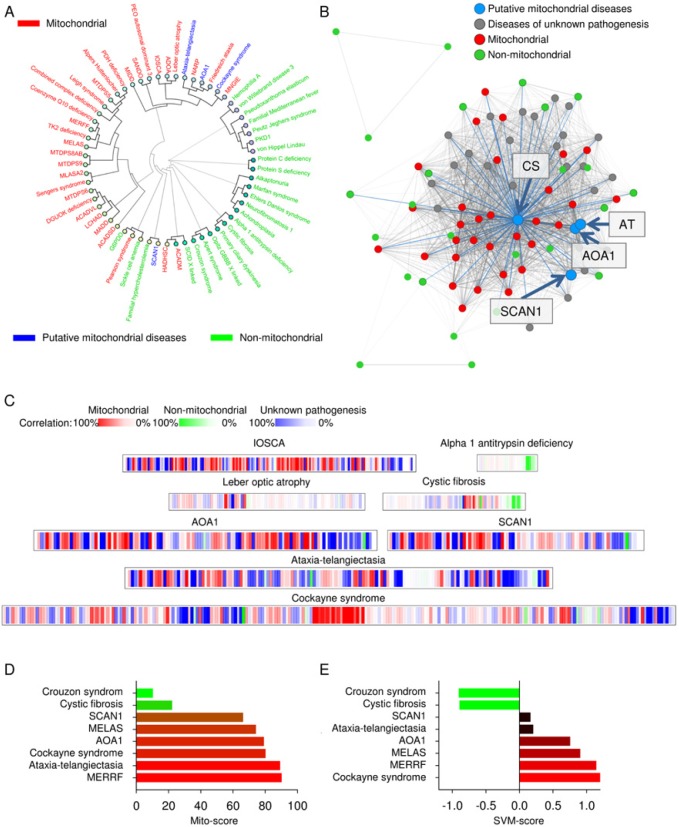
Putative mitochondrial diseases are identified using www.mitodb.com (**A**) The clustering of several diseases of unknown pathogenesis with recently recognized mitochondrial dysfunction (blue) and mitochondrial (red) and non-mitochondrial diseases (green). AOA1: Ataxia with oculomotor apraxia 1; SCAN1: Spinocerebellar ataxia with axonal neuropathy 1. (**B**) A representation of how the putative mitochondrial diseases (blue) associate within the disease network. Each dot represents a disease and the closer two diseases are connected the shorter the distance between them. Mitochondrial diseases: red; non-mitochondrial diseases: green. (**C**) The mitochondrial barcode of a number of diseases. Each bar represents a clinical parameter that is shared with another disease in the database. Red is mitochondrial diseases, green is non-mitochondrial and blue is diseases of unknown pathogenesis. The tint is given by the percentage of patients that are affected in the disease tested multiplied by the percentage of patients that are affected in the disease in the database that shares the parameter. IOSCA: infantile onset spinocerebellar ataxia (**D**) The mito score of the putative mitochondrial diseases and two bona fide mitochondrial (MERFF and MELAS) and two non-mitochondrial (cystic fibrosis and Crouzon syndrome) diseases. (**E**) The SVM score of the tested diseases and two mitochondrial (MERFF and MELAS) and two non-mitochondrial (cystic fibrosis and Crouzon syndrome) diseases.

With this established database we proceeded to develop tools to test diseases for potential mitochondrial involvement. We started by implementing hierarchical clustering algorithms using the coding languages html, javascript and php. The phenotype of a disease is entered under the “Test-disease” tab on mitodb.com by adding the prevalence of all the observed parameters. After the analysis has been completed a circular graphic illustrating (dendrogram) how the diseases cluster with each other will be generated. The connecting lines (branches of the tree) illustrate how similar two diseases are. As can be seen in figure 2A there is substantial separation of mitochondrial (red) and non-mitochondrial (green) diseases using this approach. To further visualize how the clinical parameters are interconnected we generated a disease network tool (Figure 2B) showing the association between diseases. Each dot represents a disease and each line represents a shared parameter, with the distance between the dots and the thickness of the line representing the degree of similarity (See supplementary equations). Interestingly, a central tightly associated hub of mitochondrial diseases formed with the non-mitochondrial diseases loosely associated in the periphery (Figure 2B). This is caused by the lack of overlapping parameters seen in the non-mitochondrial group compared with the highly overlapping parameters seen in the mitochondrial. Due to the substantial complexity we recommend investigat-ing this network online on www.mitodb.com, where different thresholds can be applied to selectively visualize the most common connections. To better depict the correlation between diseases we developed a mitochondrial barcode-algorithm that shows which parameters are found in other diseases in the database (Figure 2C). Each vertical colored bar represents a disease that shares a clinical parameter with the disease being tested. The tint is given by the prevalence of the parameter in the disease tested, multiplied by the prevalence in the disease in the database that shares the parameter. Thus, the barcode appears more red with increasing mitochondrial features and more green or blue with fewer or no mitochondrial features. The list of shared clinical parameters for a tested disease can be found online underneath the barcode.

### The quantitative determination of a mitochondrial phenotype using mitodb.com

To get more quantitative results we developed a tool assigning a mito-score to a disease from 0-100 with a score above 50 indicating a larger overlap with mitochondrial diseases than non-mitochondrial diseases (See supplementary equations). This method gave consistent results with mitochondrial diseases scoring high and non-mitochondrial scoring low (Figure 2D).

We next developed a machine learning algorithm to separate diseases based on clinical parameters and to get a quantitative score. Briefly, we chose a model of supervised learning, a support vector machine (SVM), that is ideal for investigating complicated datasets. This type of machine learning has been used to analyze various datasets including domain mapping of amino acid sequences in proteins and facial recognition in pictures on the web. In this method, the clinical pattern of the mitochondrial diseases and the pattern of the non-mitochondrial diseases are fed to a computer that will learn from these training examples and subsequently predict whether a tested disease may show mitochondrial involvement. A negative score reflects a likely non-mitochondrial disease while a positive score indicates a mitochondrial disease (See methods). We implemented several variations of the SVM, one unfiltered taking all parameters in the database into account and various filtered ones using only parameters displaying a cumulative prevalence of 25%, 50%, 75%, 100% or 200%. A cumulative prevalence of 25% is defined as one disease with a 25% prevalence of a parameter or five diseases with a 5% prevalence each. Thus, with increasing percentages progressively more common clinical parameters will be the only ones considered by the SVM. Overall, the performance of the various classifiers was excellent with 100% separation and a correlation coefficient (measured by cross-validation) around 0.85, with the 75% filtered version yielding a marginally better correlation coefficient of 0.87 (See supplementary equations). The great performance of all the SVM's show that the method is robust, and that the prevalence of the clinical parameters can be used to indicate whether a disease show mitochondrial involvement. To keep the data as unbiased as possible we chose the unfiltered version as our standard SVM algorithm (Figure 2E, and supplementary equations).

### The bioinformatics tools reveal mitochondrial dysfunction in ataxia-telangiectasia, Cockayne syndrome and others

Recent studies that have shown mitochondrial dysfunction in ataxia-telangiectasia [17] , AOA1 [16], SCAN1 [15] and Cockayne syndrome [14, 20]. Interestingly, these diseases clustered tightly with the mitochondrial diseases, grouped with mitochondrial disease in the disease network and displayed a relatively high SVM and mito-score (Figure 2A-E). This indicated that our tools could be used to investigate mitochondrial dysfunction in diseases of unknown pathogenesis simply by comparing the clinical parameters with the parameters in our database.

### Aging and some progerias display mitochondrial dysfunction

**Figure 3:**
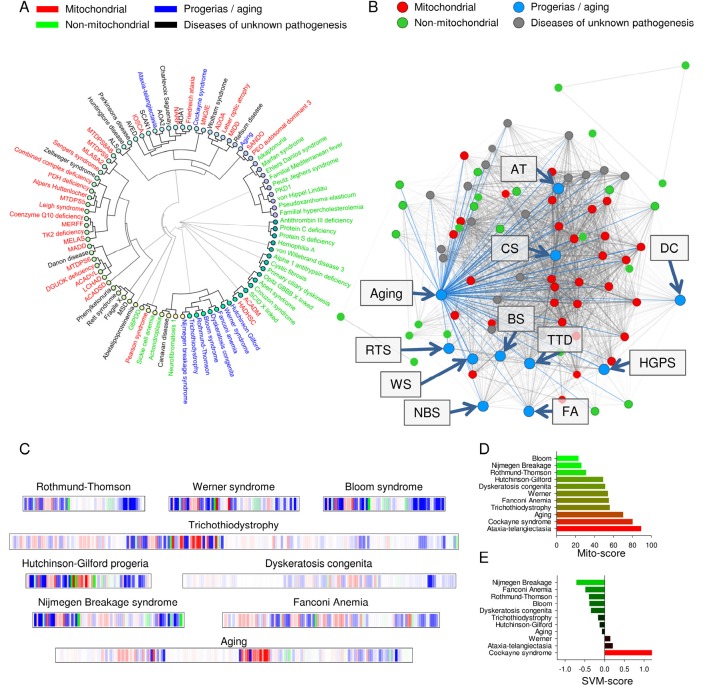
Normal aging and some accelerated aging disorders display phenotypical similarities to mitochondrial diseases (**A**) Clustermap using uncentered similarity and average linkage of all the diseases in the database. Blue represents aging and the accelerated aging disorders. (**B**) A representation of how aging and the progerias (blue dots) associate within the disease network. (**C**) Mitochondrial barcode of some accelerated aging disorders. (**D**) The mito score of the tested diseases. (**E**) The SVM score of the tested diseases.

Many diseases with unknown pathogenesis were strongly associated with mitochondrial pathology (Figure 3A and supplementary table 2). Because a close correlation between aging and mitochondrial dysfunction has been proposed, we next tested whether diseases characterized by accelerated aging shared clinical parameters with the mitochondrial diseases in the database. Of all the segmental progerias tested, Cockayne syndrome and ataxia-telangiectasia correlated tightly with mitochondrial pathology in the dendrogram, while Werner syndrome, Bloom syndrome, Rothmund-Thomson syndrome, trichothiodystrophy, dyskeratosis congenita, Fanconi anemia, Nijmegen breakage syndrome and Hutchinson-Gilford progeria formed an independent cluster (Figure 3A). This was corroborated by the finding in the disease network that Cockayne syndrome and ataxia-telangiectasia appeared to lie deeper in the mitochondrial hub compared to the other progerias (Figure 3B). When looking at the barcode for Cockayne syndrome and ataxia-telangiectasia, these diseases also showed a predominantly red impression while the other progerias where less red (Figure 2C and Figure 3C). The SVM and mito-score supported all these results (Figure 2D and E and figure 3D and E). The association between the mitochondrially associated progerias, Cockayne syndrome and ataxia-telangiectasia, was related to the high degree of neurological involvement in these diseases, since removing the neuronal parameters led these diseases to cluster with the other progerias (Supplementary figure 7).

**Figure 4:**
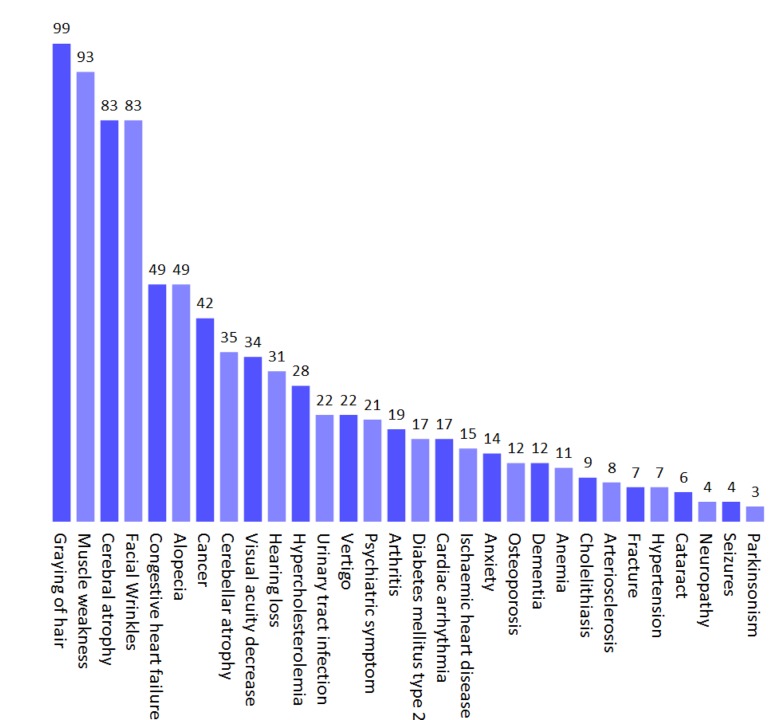
The phenotype of normal human aging based on published studies Values represent the prevalence (%) of a given parameter.

We finally investigated if normal human aging was associated with mitochondrial pathology. A number of important papers were found to describe the phenotype of normal human aging as accurately as possible (Figure 4) [21-30]. By entering the parameters of aging an interesting picture emerged. Normal aging seemed to cluster with the mitochondrial diseases where the mitochondrial segmental progerias also clustered indicating a potential mitochondrial phenotype (Figure 3A-C). Reflecting this, the barcode showed a mitochondrial imprint that was corroborated by a high mito-score of 70. However, the SVM was inconclusive reflected by a score of around 0. Interestingly, using the 200% filtered SVM, aging received a score of 1.46, indicating a probable mitochondrial impact when considering only very common clinical parameters. It therefore seems likely that some features of aging, eg. cancers, may be independent of mitochondria while others, eg. neurological features, are closely correlated with mitochondrial health.

## DISCUSSION

The use of bioinformatics in medical pattern recognition is a powerful tool ideally suited to support clinicians and scientists when investigating large datasets. Mitochondrial diseases represent a bioinformatically ideal group of pathologies because they are etiologically well defined while presenting a complex clinical picture. Here we introduce a novel mitochondrial database and a unique array of useful tools to investigate potential mitochondrial involvement in diseases of unknown pathogenesis. We have placed the database online to ensure the highest usability and a continuous evolution based on the addition of new diseases and revisions of clinical parameters. Mitochondrial diseases are highly complex involving multiple organ systems with a large variability in age of onset and clinical severity. It is therefore not surprising that there is a very substantial lag phase in disease onset and time of diagnosis in many of these diseases. We believe that the tools we have presented here can be helpful in the diagnosis of these diseases potentially allowing earlier treatments for this group of severe disorders.

The neuropathology observed in some of the accelerated aging disorders seems to drive the association with mitochondrial diseases in the database. This is not unexpected since mitochondrial dysfunction has been implicated in many age related neurological disorders such as Parkinson's and Huntington's disease. In recent years increasing evidence of mitochondrial involvement has emerged in these diseases. For Parkinson's disease mutations in PINK1 and parkin have implicated the degradation of damaged mitochondria through autophagy as part of the pathogenesis [31]. Interestingly, this pathway has also been suggested to be defective in Huntington's disease perhaps indicating a common pathogenesis [32]. In support of a mitochondrial dysfunction in these neurodegenerative disorders the basal ganglia are often affected in mitochondrial diseases such as Leigh syndrome and Neuropathy, Ataxia and Retinitis Pigementosum (NARP syndrome) [33, 34]. Indeed, Parkinson's disease receives a mito-score of 100 while Huntington's disease receives a mito-score of 99 and both have a positive SVM score.

In addition to the clinical uses the tools presented herein could be valuable for scientists investigating the cause of diseases of unknown pathogenesis. Indeed, using the tools, we corroborate the findings recently published for ataxia-telangiectasia and Cockayne syndrome [14, 17]. We further show tight associations between mitochondrial pathology and AOA1 and SCAN1, two diseases recently shown to have mitochondrial defects [15, 16]. In this context, it is important to point out that these tools do not specify whether a disease is primarily mitochondrial or if the exposed dysfunction stems from a secondary unknown mechanism.

According to our hypothesis premature aging disorders as well as normal aging should share features with mitochondrial disease. This seems to be partially true. Based on the hierarchical clustering it appears that two distinct types of accelerated aging disorders exist. One type consisting of Werner syndrome, Bloom syndrome, Rothmund-Thomson syndrome, trichothiodystrophy, dyskeratosis congenita, Fanconi anemia, Nijmegen breakage syndrome and Hutchinson-Gilford progeria form a separate cluster. For these disorders the common molecular denominator involves chromosome instability in relation to DNA replication, repair of double stranded DNA breaks and telomere maintenance [35-37]. The second group of accelerated aging disorders consists of Cockayne syndrome and ataxia-telangiectasia. These two disorders are characterized by a substantial neurological phenotype that drives the clustering with the mitochondrial diseases. Interestingly, normal aging itself, seems to associate closer with the mitochondrial diseases that the non-mitochondrial diseases perhaps corroborating the mitochondrial theory of aging.

Noticeably, during the development of the database we encountered a number of syndromes of unknown pathology displaying strong correlation with mitochondrial diseases (Supplementary table 2). Several of these diseases exhibit severe neuropathology supporting the idea that the central nervous system is particularly prone to mitochondrial dysfunction [10, 38]. Disorders of this nature would thus be prime targets for further investigation and potentially for treatment with drugs known to augment mitochondrial function.

## METHODS

### Hierarchical clustering

Hierarchical clustering was implemented using common similarity measures and linkage methods (See supplementary equations) [18, 39]. For each clustering the cophenetic correlation is calculated as a measure of how well the clustering was achieved revealing uncentered similarity and average linkage producing the best result (see supplementary figure 9 and supplementary equations). Other similarity metrics and linkage methods can be chosen online by clicking “advanced” under the “test-disease” tab on www.mitodb.com.

### Support vector machine (SVM)

The support vector classifier was created by exporting the symptom-vectors of the known mitochondrial and non-mitochondrial diseases. The symptom-vectors were processed with the statistical program “R” using the “e1071” package (http://cran.r-project.org/web/packages/e1071/index.html), which is based on the library “libSVM” to train the SVMs. The SVMs were trained using a “linear kernel” and “eps-regression”, and displays (using 11-fold cross-correlation) a total mean squared error of 0.32, a 0.84 correlation coefficient, and 100% separation. The parameters of the SVMs were then exported from R and the classifier functions for the web-page was created (with php) using these parameters.

Further methods can be found in the supporting materials.

## SUPPORTING INFORMATION


