# Supplementary material for: A novel diagnostic tool reveals mitochondrial pathology in human diseases and aging
Source: Aging (Albany NY). 2013 Mar 24;5(3):192–208. doi: 10.18632/aging.100546 (PMC3629291; doi:10.18632/aging.100546)
Supplement: Supplementary file 1 [file aging-05-192-s001.pdf]

SUPPORTING INFORMATION

Supplementary Figure 1. The publication dates of the references used in the database.

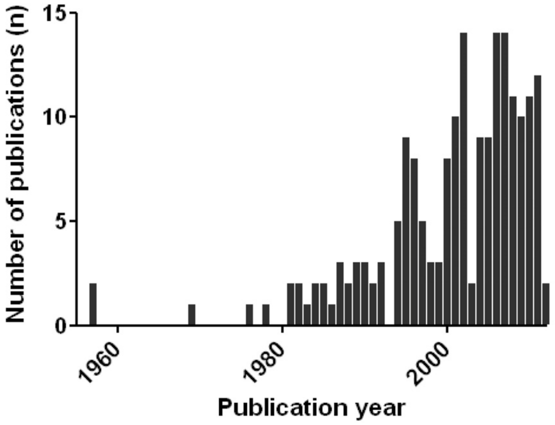

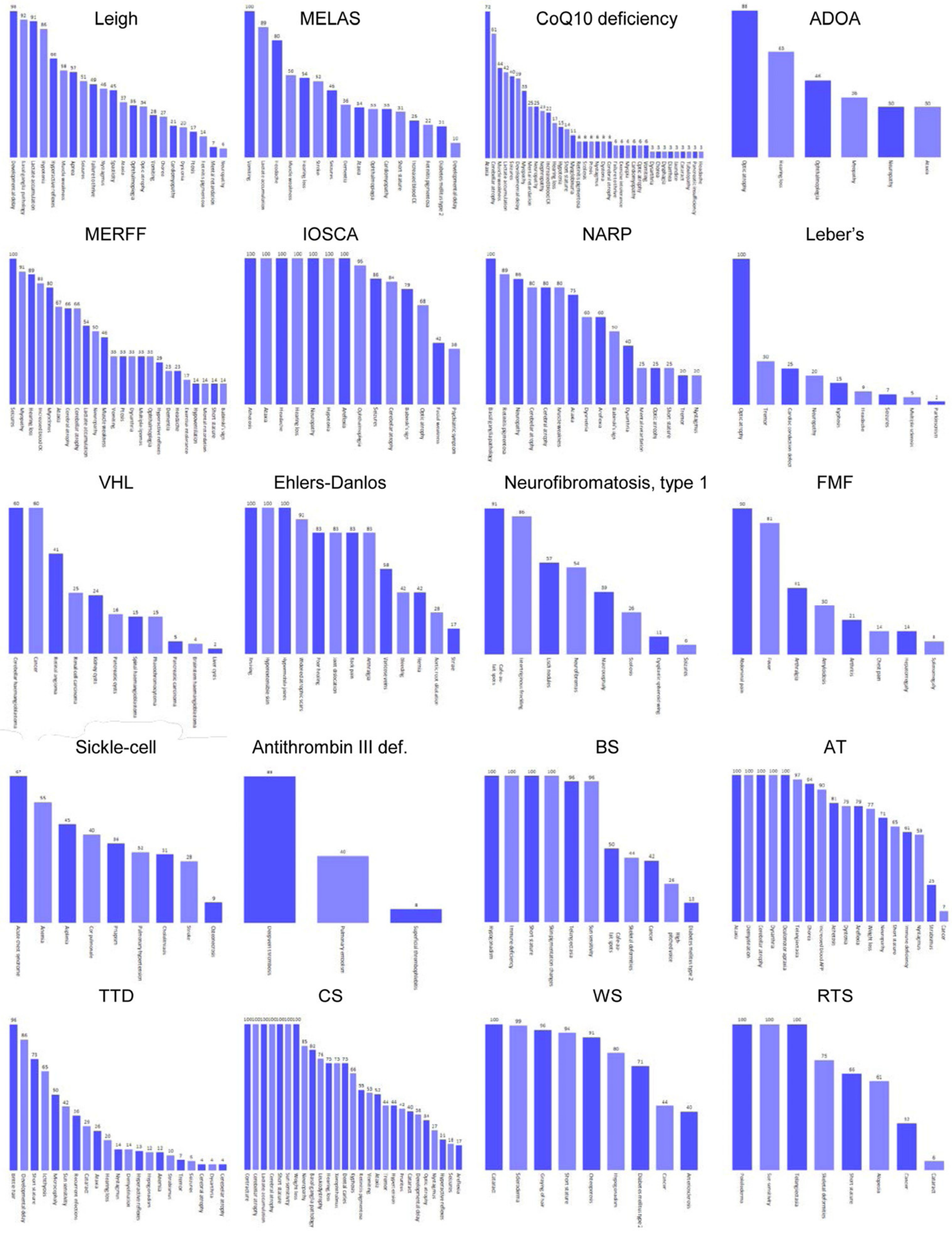

Supplementary Figure 2. Visual representation of some diseases and their clinical parameters as seen in the database. See supplementary table 1 for abbreviations.

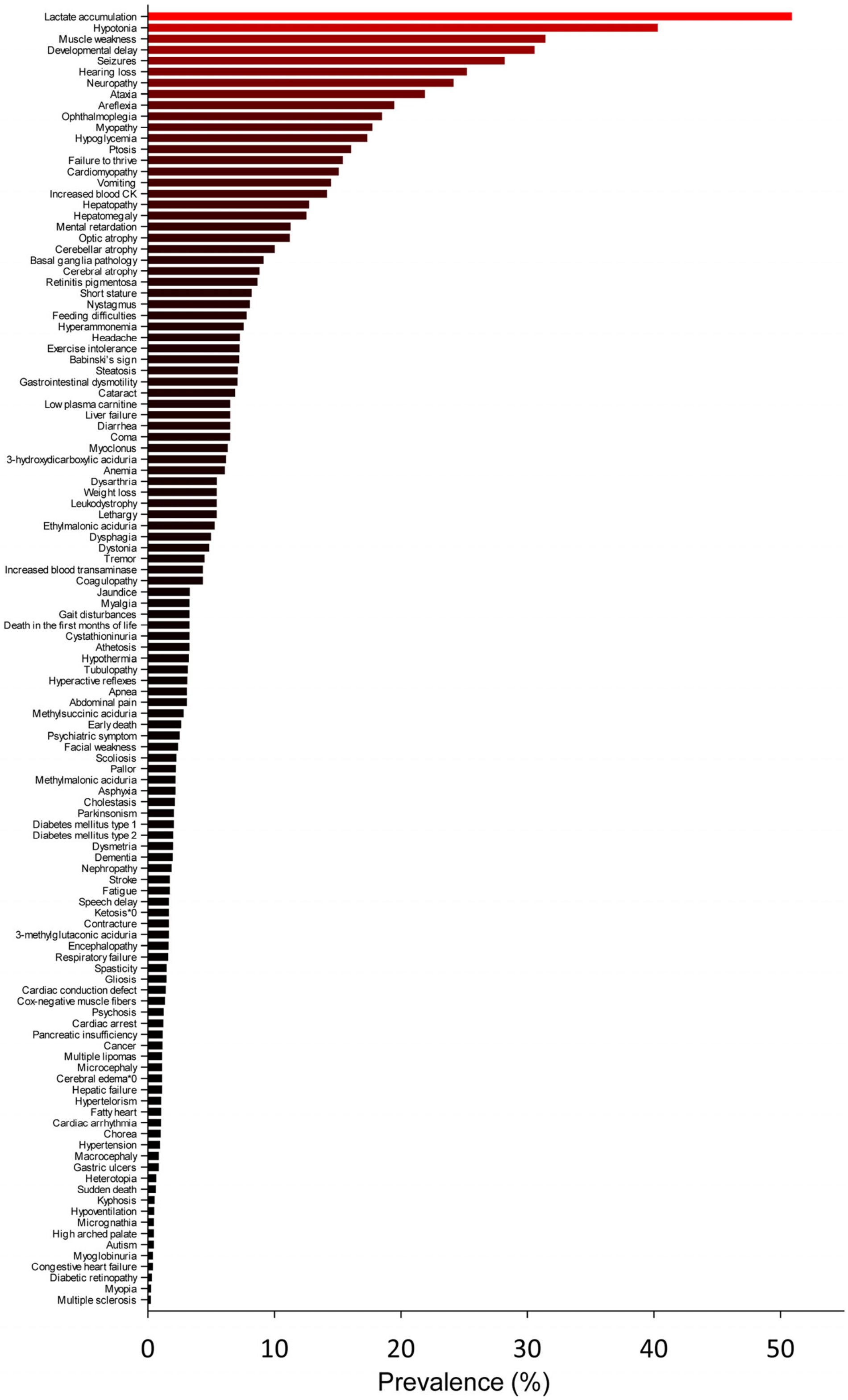

Supplementary Figure 3 Mitochondrial diseases in the database have a defined clinical spectrum. The average prevalence of clinical parameters across all the non-mitochondrial diseases in the database. Inset: Close-up of the top-20 clinical parameters seen in non-mitochondrial diseases.

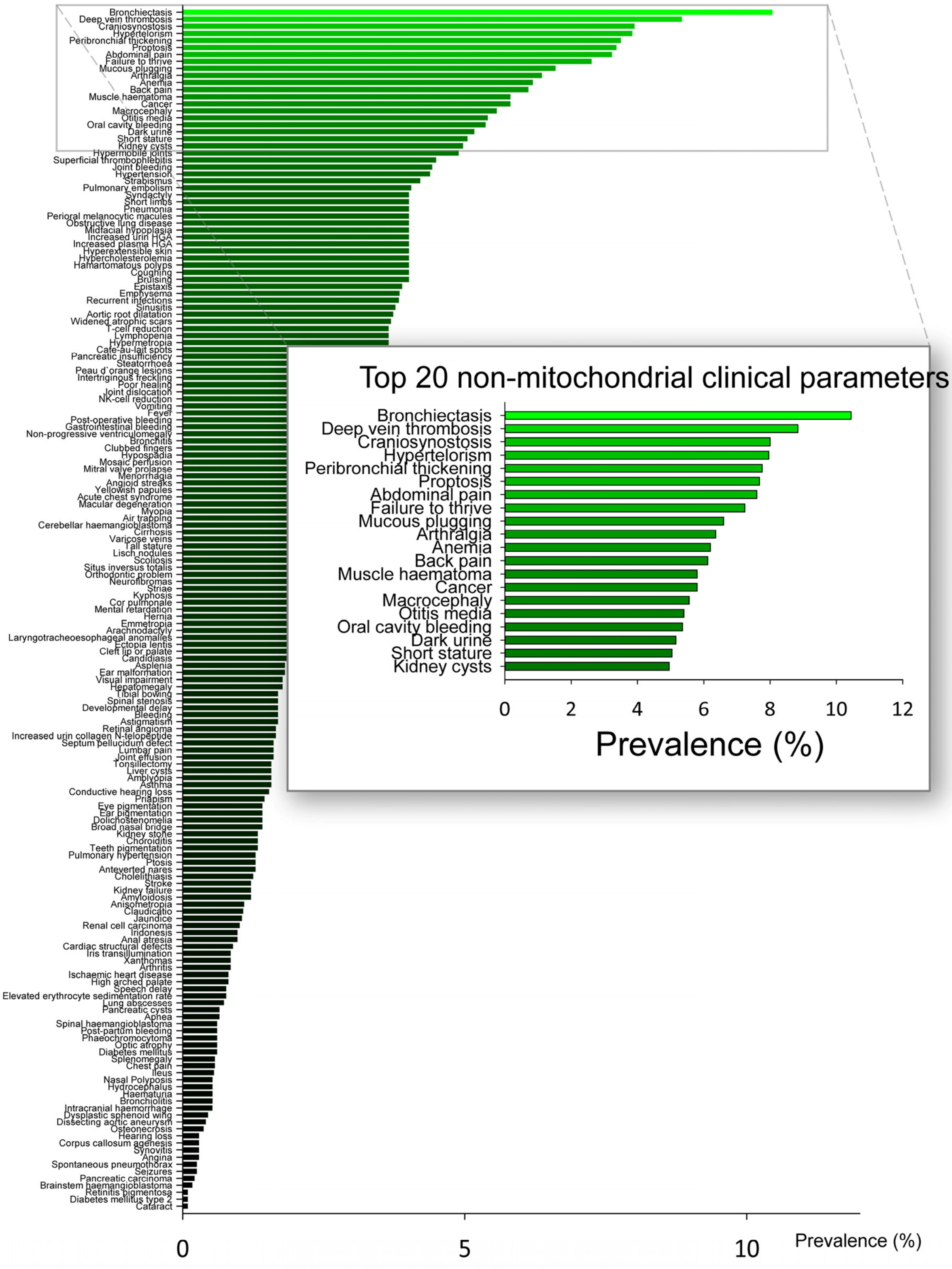

Supplementary Figure 4. Non-mitochondrial diseases in the database do not have a defined clinical spectrum. The average prevalence of clinical parameters across all the non-mitochondrial diseases in the database. Inset: Close-up of the top-20 clinical parameters seen in non-mitochondrial diseases.

A

B

C

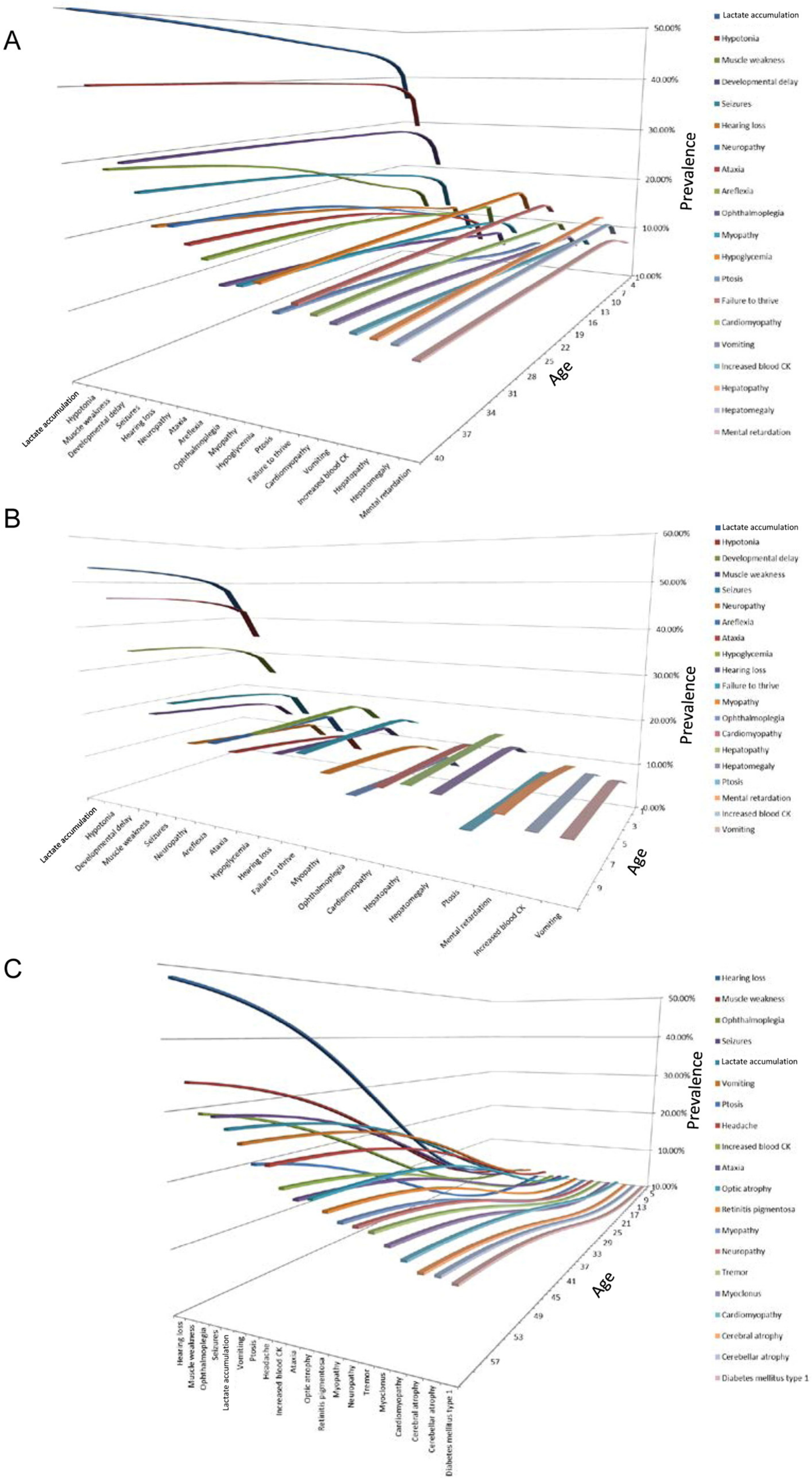

Supplementary Figure 5. The approximated age of onset of parameters in mitochondrial diseases. (**A**) The average prevalence of clinical parameters across all the mitochondrial diseases as a function of age. (**B**) The average prevalence of clinical parameters across the mitochondrial diseases with an onset before age 20 as a function of age. (**C**) The average prevalence of clinical parameters across the mitochondrial diseases with an onset after age 20 as a function of age.

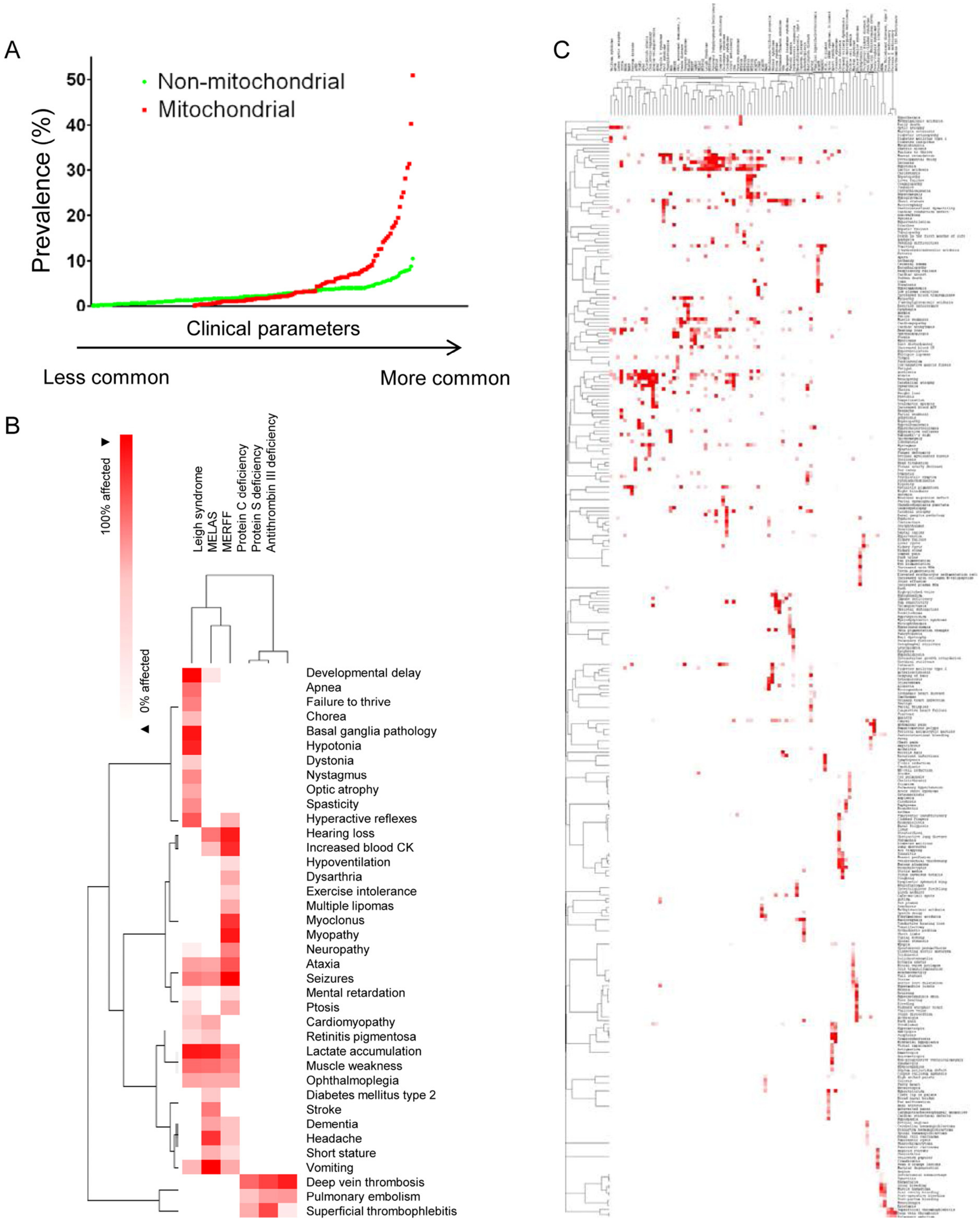

Supplementary Figure 6. Mitochondrial diseases cluster well with each other. (**A**) An overlay of the prevalence of clinical parameters seen in mitochondrial and non-mitochondrial diseases in the database. Note: The clinical parameters in the mitochondrial and non-mitochondrial diseases do not correspond to each other. For the non-mitochondrial clinical parameters see supplementary figures. (**B**) An example of the clustering of diseases in the database using uncentered similarity metrics and average linkage. The diseases are listed across the top, the dendrogram below, with the clinical parameters denoted on the right. The tint of the square represents the prevalence of the parameter in the given disease. (**C**) The complete clustermap of all the diseases in the database. Each square represents a referenced parameter that can be found online on www.mitodb.com.

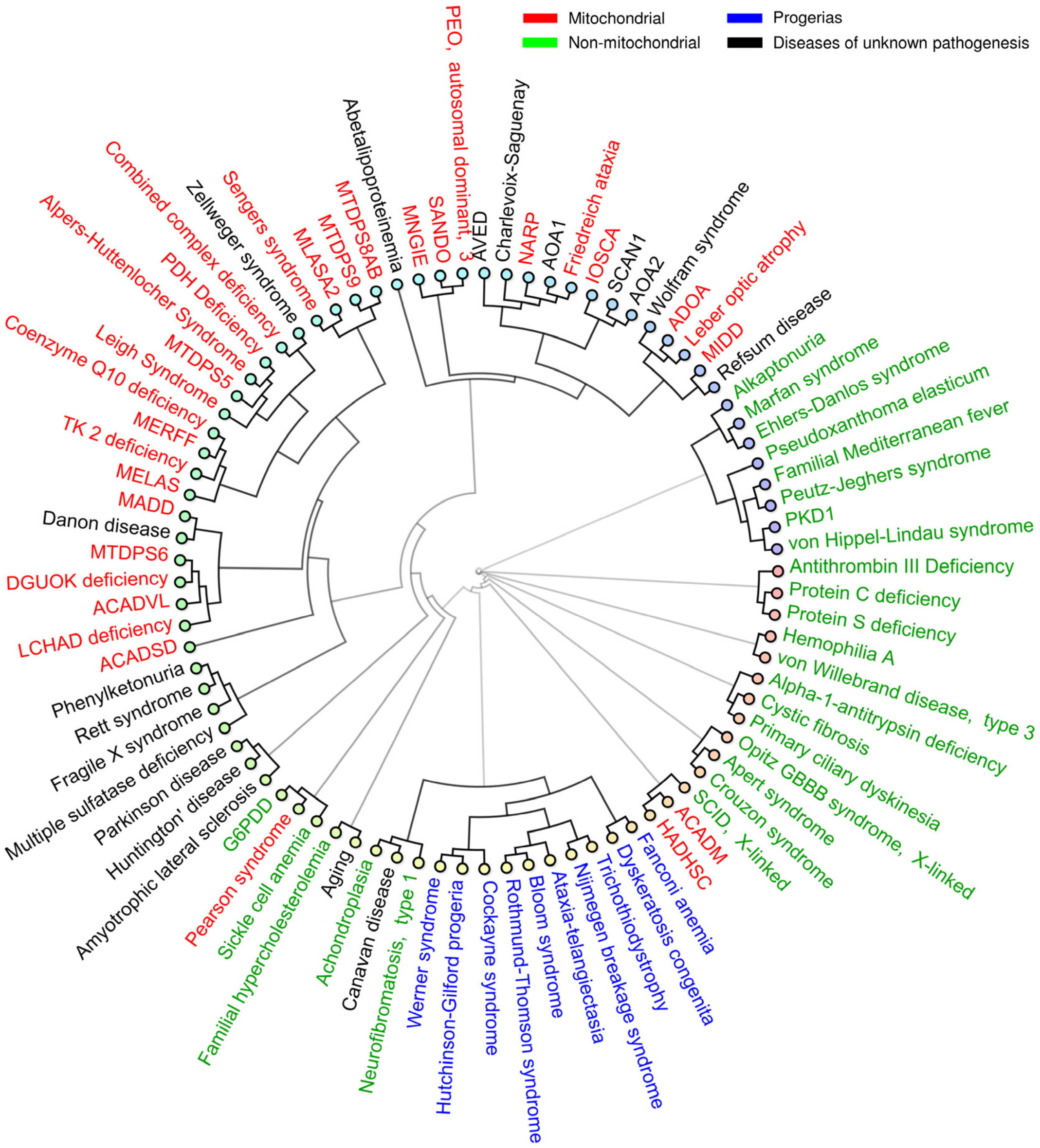

Supplementary Figure 7. Removing neuronal parameters allow the accelerated aging disorders to cluster together. Clustermap using uncentered similarity and average linkage of all the diseases in the database. Blue represents the accelerated aging disorders where the neuronal parameters have been removed.

Supplementary table 1 **A list of the diseases currently in the database and their mean age of onset.**

Mitochondrial	Abbreviation	OMIM		Age of onset	SD	PMID
Autosomal dominant optic atrophy	ADOA	165500		7.21	3.57	20417570
Medium-Chain Acyl-CoA Dehydrongenase Deficiency	ACADM	201450		1.83	0.72	6646897
Short Chain Deficiency of ACYL-CoA Dehydrogenase	ACADSD	201470		1.55	2.23	18054510
Very Long Chain Deficiency of ACYL-CoA Dehydrogenase	ACADVL	201475		0.14	0.15	7769092
Alpers-Huttenlocher Syndrome	MTDPS4A	203700		0.11	0.23	20142534
Sengers syndrome		212350		0.28	0.35	16736096
Friedreich ataxia		229300		15.50	8.00	8815938
3-Hydroxyacyl-CoA Dehydrogenase Deficiency	HADHSC	231530		0.22	0.29	10347277
Multiple Acyl-CoA Dehydrogenase Deficiency	MADD	231680		15.94	13.52	17977044, 20370797
Encephalomyopathy with methylmalonic aciduria	MTDPS9	245400		0.00	0.00	17668387
Deoxyguanosine kinase deficiency	DGUOK deficiency	251880		0.11	0.12	16908739
Leigh Syndrome		256000		0.94	1.04	8602753
Mitochondrial DNA depletion syndrome 6	MTDPS6	256810		0.57	0.36	16909392
Infantile onset spinocerebellar ataxia ,MTDPS7	IOSCA	271245		1.20	0.27	8133312
Maternally transmitted diabetes and deafness syndrome	MIDD	520000		34.60	13.90	11329229
Leber optic atrophy		535000		24.34	13.98	7735876
Mitochondrial myopathy, encephalopathy, lactic acidosis, and stroke-like episodes	MELAS	540000		20.33	14.53	11708999
Myoclonic Epilepsy associated with Ragged Red Fibers	MERFF	545000		34.67	15.06	21303704
Neuropathy Ataxia and Retinitis Pigmentosa	NARP	551500		14.25	14.01	20953793
Pearson syndrome		557000		0.55	0.71	7581370
Mitochondrial neurogastrointestinal encephalopathy	MNGIE	603041		17.58	11.03	21933806
Coenzyme Q10 deficiency		607426		2.99	3.96	17442627
Sensory ataxic neuropathy, dysarthria and ophthalmoparesis	SANDO	607459		14.33	6.19	15824347
Pyruvate Dehydrogenase Deficiency	PDH deficiency	608782		2.47	3.06	1327585
Long-chain 3-hydroxyacyl–coenzyme A dehydrogenase deficiency	LCHAD	609016		0.67	0.73	9003853
Progressive external ophthalmoplegia, autosomal dominant, 3	PEO, autosomal dominant, 3	609286		40.94	15.14	20479361
Thymidine kinase 2 deficiency	TK2 deficiency	609560		0.61	0.55	18819985
Encephalomyopathy with methylmalonic aciduria	MTDPS5	612073		0.12	0.15	17301081
Encephalomyopathy with renal tubulopathy	MTDPS8AB	612075		0.04	0.05	17486094
Myopathy, mitochondrial progressive, with congenital cataract, hearing loss, and developmental delay	Combined complex deficiency	613076		0.00	0.00	19409522
Myopathy, Lactic acidosis, and Sideroblastic Anemia 2	MLASA2	613561		1.10	1.65	20598274
**Non mitochondrial**						
Achondroplasia		100800		0.00	0.00	690757
Apert syndrome		101200		0.00	0.00	7645606
Crouzon syndrome		123500		0.00	0.00	15885794
Ehlers-Danlos syndrome		130000		12.67	2.08	17762038
Familial hypercholesterolemia		143890		29.00	18.00	227426
Marfan syndrome		154700		11.40	3.95	9059160
Neurofibromatosis, type 1		162200		14.90	12.90	18277076
Polycystic kidney disease 1	PKD1	173900		54.00	19.00	22367170
Peutz-Jeghers syndrome		175200		19.00	9.45	20126809
Congenital protein C deficiency		176860		24.10	11.90	20126809
von Hippel-Lindau syndrome	VHL	193300		26.25	12.53	8929948
Alkaptonuria		203500		55.38	60.52	21927854
Cystic fibrosis, CF		219700		3.70	7.20	12001283
Primary ciliary dyskinesia		244400		21.04	13.59	9387968
Familial Mediterranean fever		249100		5.50	3.40	9266193
Pseudoxanthoma elasticum		264800		30.50	16.50	17693525
von Willebrand disease, type 3		277480		3.00	6.71	19473418
Opitz GBBB syndrome, X-linked		300000		0.00	0.00	15558842
Severe combined immunodeficiency, X-linked	SCID, X-linked	300400		2.38	1.70	8185357
Glucose-6-phosphate dehydrogenase deficiency	G6PDD	305900		1.79	2.78	7110809
Hemophilia A	HEMA	306700		0.50	0.50	10650861
Sickle cell anemia		603903		2.18	1.19	22224796
Protein S deficiency		612336		23.88	5.84	2959350
Antithrombin III Deficiency		613118		31.67	14.03	1489375
Alpha-1-antitrypsin deficiency		613490		42.73	16.79	309708
**Unknown pathogenesis**						
Aging		0				
Amyotrophic lateral sclerosis	ALS	105400		54.62	10.90	17296839
Dyskeratosis congenita	DC	127550		29.10	20.23	21436073
Huntington disease		143100		42.30	14.90	11574110
Parkinson disease		168600		62.20	10.60	22362919
Hutchinson-Gilford progeria	HGPS	176670	est:	2.00	2.00	18256394
Abetalipoproteinemia		200100		14.67	5.65	10679949
Ataxia-telangiectasia	AT	208900		2.32	1.20	1377828
Ataxia with oculomotor apraxia type 1	AOA1	208920		5.53	1.30	21465257
Bloom syndrome	BS	210900	est:	0.00	0.00	16763388
Zellweger syndrome		214100		0.00	0.00	9818927
Cockayne syndrome	CS	216400		3.29	2.54	17092472
Wolfram syndrome		222300		4.14	1.95	21968327
Fanconi anemia	FA	227650		6.34	3.97	12393516
Nijmegen breakage syndrome	NBS	251260		0.69	0.05	15033202
Phenylketonuria		261600		3.76	7.32	13452670
Refsum disease		266500		19.73	9.65	2433405
Rothmund-Thomson syndrome	RTS	268400		1.64	1.99	13393794
Charlevoix-Saguenay	ARSACS	270550		5.50	1.60	16961075
Canavan disease		271900		0.81	2.18	9568915
Multiple sulfatase deficiency	MSD	272200		1.00	0.82	18509892
Ataxia with selective vitamin E deficiency	AVED	277460		13.25	8.25	15300460
Werner syndrome	WS	277700		34.71	8.00	16673358
Danon disease		300257		12.43	3.82	19318653
Fragile X syndrome		300624		3.10	2.18	22134579
Rett syndrome		312750		4.70	3.70	20728410
Trichothiodystrophy	TTD	601675		2.75	0.35	11709541
Ataxia with oculomotor apraxia type 2	AOA2	606002		14.60	3.40	19696032
Spinocerebellar Ataxia with Axonal Neuropathy	SCAN1	607250		13.67	1.15	12244316

*Some diseases are represented with the abbreviated titles in the main figures.

Supplementary Figure 8. Uncentered similarity metrics and average linkage yields the best clustering. The cophenetic correlation coefficient (see supplementary equations) plotted using different weighting of uncentered and centered similarity and average and centroid linkage and clustering of all the diseases or only mitochondrial and non-mitochondrial diseases.

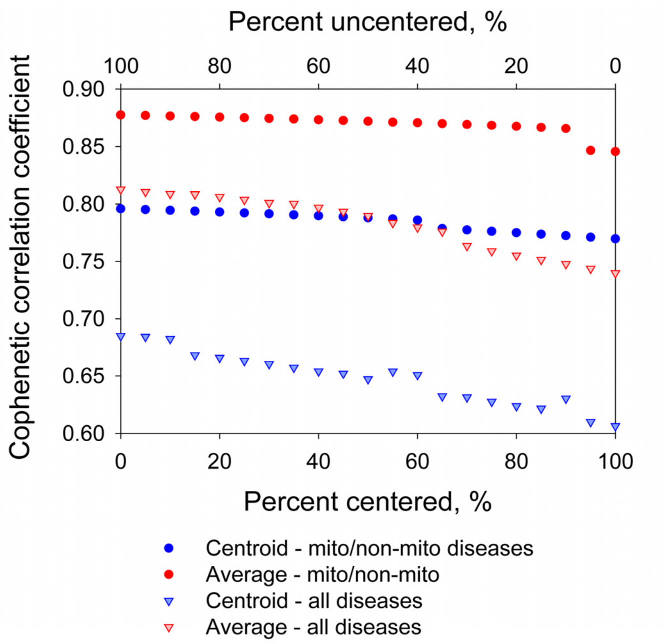

Supplementary table 2 **Some diseases of unknown pathogenesis display a mitochondrial phenotype.**

Disease	OMIM ID	Mito clustering?	Mito score	SVM	Mitochondrial?
ALS	105400	No	100	0.23	++
Huntington's disease	143100	No	99	0.17	++
Parkinson's disease	168600	No	100	0.26	++
Abetalipoproteinemia	200100	No	53	-0.18	-
Zellweger syndrome	214100	Yes	75	0.37	+ + +
Wolfram syndrome	222300	Yes	94	1.09	+ + +
Phenylketonuria	261600	Yes	59	-0.26	+
Refsum disease	266500	Yes	96	0.33	+ + +
Charlevoix-Saguenay	270550	Yes	100	0.4	+ + +
Canavan disease	271900	No	67	0.74	+ +
Multiple sulfatase deficiency	272200	Yes	73	-1.05	+
AVED	277460	Yes	92	0.38	+ + +
Danon disease	300257	Yes	83	1.65	+ + +
Fragile X syndrome	300624	Yes	36	0.07	+
Rett syndrome	312750	Yes	68	0.61	+ + +
AOA2	606002	Yes	97	0.02	+ + +

The last column represents our interpretation of how strong the mitochondrial features are based on the tests we have done. Each disease receives a +/- sign for clustering with mitochondrial diseases, scoring more than 50 in the mito-score or receiving a positive SVM-score.
